# Epithelial Cells in 2D and 3D Cultures Exhibit Large Differences in Higher-order Genomic Interactions

**DOI:** 10.1016/j.gpb.2020.06.017

**Published:** 2021-02-23

**Authors:** Xin Liu, Qiu Sun, Qi Wang, Chuansheng Hu, Xuecheng Chen, Hua Li, Daniel M. Czajkowsky, Zhifeng Shao

**Affiliations:** 1 State Key Laboratory for Oncogenes and Bio-ID Center, School of Biomedical Engineering, Shanghai Jiao Tong University, Shanghai 200240, China; 2 Shanghai Center for Systems Biomedicine, Shanghai Jiao Tong University, Shanghai 200240, China; 3 Translational Medical Center for Stem Cell Therapy & Institute for Regenerative Medicine, Shanghai East Hospital, School of Life Science and Technology, Shanghai Key Laboratory of Signaling and Disease Research, Tongji University, Shanghai 200092, China

**Keywords:** 3D culture, *In situ* Hi-C, Chromosome conformation, Compartment, TAD

## Abstract

Recent studies have characterized the genomic structures of many eukaryotic cells, often focusing on their relation to gene expression. However, these studies have largely investigated cells grown in 2D cultures, although the transcriptomes of 3D-cultured cells are generally closer to their *in vivo* phenotypes. To examine the effects of spatial constraints on **chromosome conformation**, we investigated the genomic architecture of mouse hepatocytes grown in 2D and **3D cultures** using ***in situ* Hi-C**. Our results reveal significant differences in higher-order genomic interactions, notably in **compartment** identity and strength as well as in topologically associating domain (**TAD**)–TAD interactions, but only minor differences are found at the TAD level. Our RNA-seq analysis reveals an up-regulated expression of genes involved in physiological hepatocyte functions in the 3D-cultured cells. These genes are associated with a subset of structural changes, suggesting that differences in genomic structure are critically important for transcriptional regulation. However, there are also many structural differences that are not directly associated with changes in gene expression, whose cause remains to be determined. Overall, our results indicate that growth in 3D significantly alters higher-order genomic interactions, which may be consequential for a subset of genes that are important for the physiological functioning of the cell.

## Introduction

The importance of genome structure on the functioning of the genome has now been well established [1], [2], [3], [4], [5]. In recent years, perhaps foremost among the methods used to this end are those based on the chromosome conformation capture techniques, most notably *in situ* Hi-C [6]. Such work has revealed a hierarchy of structural features, from locally compact, so-called topologically associating domains (TADs) to more distant TAD–TAD interactions, including broad multi-megabase regions called compartments that are designated as A or B, associated with transcriptionally active or inactive chromatin [7], [8]. Although the underlying mechanisms driving the formation of both TADs and compartments are still incompletely understood, their conservation among different cell types and across species suggests that they constitute fundamental components of the eukaryotic genome architecture [4], [9], [10].

One of the major focuses of previous work has been the determination of the relationship between the genome structure and gene expression [11], [12], [13], [14]. Indeed, there have been well-described changes at both the TAD and compartment levels that are significantly related to the transcriptional statuses of genes contained therein [15], [16], [17]. Still, there are recent examples of clear differences in gene expression with no detectable changes in the corresponding Hi-C data [18], [19], indicating that there can be more complex determinants of the genome structure than just the transcriptional status.

However, to date, almost all of these studies have focused on the genome structures of cultured cells, either immunological cells grown in suspension or, more commonly, adherent cells grown in two-dimensional (2D) cultures [6], [20], [21], [22]. Such systems are unquestionably powerful to identify biologically important proteins as well as the functions of these proteins in regulating chromosome structure. However, there is still the question of whether or not the overall structures of the genomes in the adherent cells as determined by 2D cultures are applicable to their *in vivo* counterparts. Cells within tissues contact other cells and extracellular matrix components in three dimensions (3D), which can affect gene expression and chromatin conformation [23].

In this regard, there are now well-established methods for culturing cells in 3D [24], [25], [26], and indeed the 3D-cultured cells exhibit transcriptomes and cellular behaviors that are more typical of cells within their physiological environment [27], [28], [29]. The overall cell shape and the nuclear shape of cells grown in 3D are also often significantly different from the same type of cells grown in 2D [30], [31]. Such differences in nuclear shape, in particular, suggest that there are likewise differences in the interactions between whole chromosomes, if not also in the structural details at the sub-chromosomal level. However, the nature of these differences and their relation to differences in transcription have not been well examined. In fact, to our knowledge, the only published Hi-C study which focused on characterizing the differences in genome structure between cells grown in 2D and 3D examined fibroblast cells [30], whose nuclear shape only differs marginally when grown in 2D or 3D (15% change in volume; see Figure S2 in [30]). This work indeed identified differences in genome structure, although at a relatively low resolution of 1 Mb, which is too low to resolve the majority of TADs (median length 185 kb [6]). However, the generality of these results, especially to cells of an epithelial origin, and details of the changes in genomic structure more locally have not yet been elucidated. Moreover, how these changes in genome structure between cells cultured in 2D and 3D relate to the changes in gene expression is also poorly documented.

Here, we examined the genome structures of mouse hepatocytes cultured under 2D and 3D conditions and their relation to gene expression using RNA sequencing (RNA-seq) and *in situ* Hi-C (at a resolution of 40 kb). Overall, we find that the 3D conformation of the genome is clearly different from the 2D conformation but, unexpectedly, only in terms of higher-order interactions, not at the local TAD-level. Nonetheless, a subset of the structural changes in the 3D-cultured cells is associated with the up-regulation of genes that are involved in typical functions of hepatocytes. Thus, growth dimension indeed influences not only cell behavior and transcriptome but also the genome structure, which appears to play a role in effectuating the physiological phenotype of the cell.

## Results

### *In situ* Hi-C of hepatocytes cultured under 2D and 3D conditions

**Figure 1:**
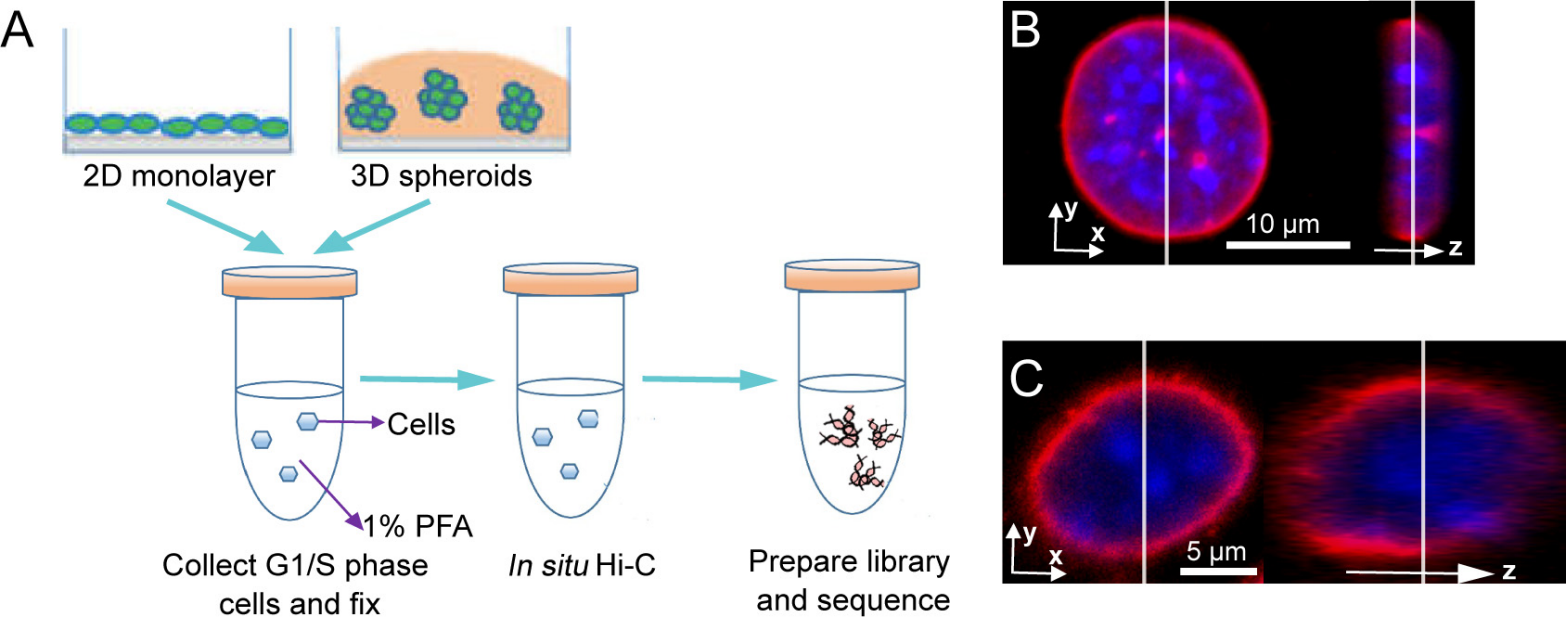
**Overview of the experiment** **A.** Workflow of the experiment. **B.** and **C.** Confocal microscopy images of the 2D-cultured (B) and 3D-cultured (C) mouse AML12 hepatocytes stained with DAPI (blue) and fluorescent antibodies for lamina B (red). PFA, paraformaldehyde.

**Supplementary Figure S1:**
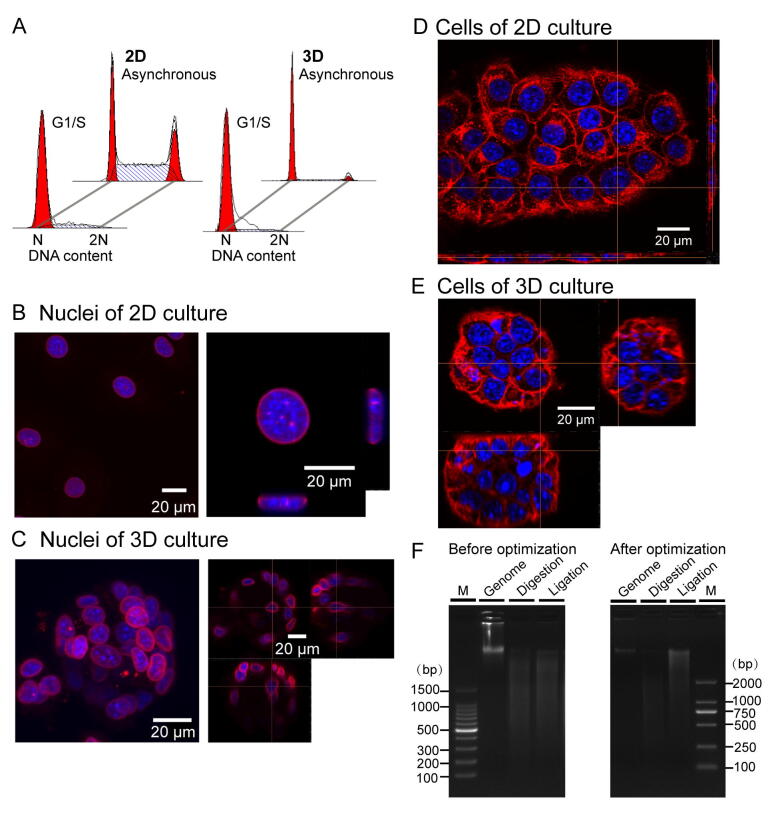
**Characterization of the 2D- and 3D-cultured AML12 cells A.** Flow cytometry of the synchronized 2D- and 3D-cultured cells. **B.** and **C.** Confocal images of the nuclei of 2D-cultured (B) and 3D-cultured (C) cells stained with DAPI (blue) and lamina B (red). **D.** and **E.** Confocal images of 2D-cultured (D) and 3D-cultured (E) cells stained with DAPI (blue) and Wheat Germ Agglutinin (red to label the cell membrane). **F.** Digestion and ligation efficiency before and after optimization of the conditions to culture cells in 3D.

To investigate the effects of growth dimension on cell growth, we performed *in situ* Hi-C on alpha mouse liver 12 (AML12) cells, a mouse hepatocyte cell line, grown in either 2D or 3D conditions (Figure 1A). For the latter, cells were grown in Matrigel-embedded 3D cultures, which contain extracellular matrix components that are important regulators of normal homeostasis and tissue phenotype [32], [33]. Since chromatin structures are known to change significantly during the cell cycle [34], we arrested the cells grown under both conditions at the G1/S boundary using hydroxyurea, an inhibitor of DNA replication [35]. The G1/S state of these cells was confirmed using flow cytometry (Figure S1A).

Consistent with previous reports, we found that the morphological features of the cells were significantly different when grown in 2D or 3D [36]. In particular, while the 2D-cultured cells grew as a single layer, the 3D-cultured cells grew as many individual 3D spheroids with extensive and multiple contacts between the cells (Figure S1B and C). Moreover, the overall cell shape and the nuclear shape of the 2D-cultured cells were significantly different from those of the 3D-cultured cells (Figure 1B and C, Figure S1D and E). Quantitatively, we found that the mean volume of the nuclei of the 2D-cultured cells was nearly twice that of the 3D-cultured cells (875 ± 71 μm^3^ and 362 ± 35 μm^3^, respectively). Such a difference was much more significant than that previously observed in fibroblast cells [30]. Owing to the influence of volume on the T4 DNA ligase efficiency, we adjusted the experimental conditions to optimize the ligation efficiency for Hi-C under our conditions (Figure S1F; see Materials and methods for details).

### Overview of the chromatin organization of hepatocytes cultured in 2D and 3D

**Supplementary Figure S2:**
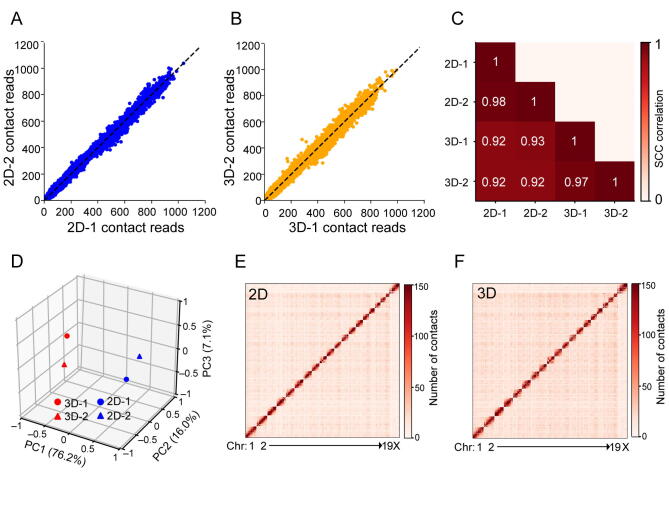
**A compendium of the sequencing data of the in situ Hi-C libraries A.** Correlation between the biological replicates of the 2D-cultured cells (SCC = 0.98). **B.** Correlation between the biological replicates of the 3D-cultured cells (SCC = 0.97). **C.** Heatmap showing the pairwise SCC correlations between all samples of the 2D- and 3D-cultured cells. **D.** PCA of the individual biological replicates of the 2D- and 3D-cultured cells. The percentages along each axis reflect the fraction of the variances. **E.** and **F.** The whole genome chromatin contact maps showing inter-chromosomal contacts of the 2D-cultured (E) and 3D-cultured (F) cells. PCA, principal component analysis.

After sequencing the Hi-C libraries, we generated 214 million and 156 million raw reads, yielding 71 million and 68 million valid paired-end reads after all filtration steps, of the 2D- and 3D-cultured cells, respectively, following a previously described protocol [37]. To evaluate the reliability of our data, we examined a biological replicate for each culture condition, and generated 195 million and 143 million raw reads which finally yielded 80 million and 61 million valid paired-end reads of the 2D- and 3D-cultured cells, respectively (Table S1). Both cultured cells were highly correlated with their corresponding biological replicate with the stratum-adjusted correlation coefficient (SCC) of 0.98 for 2D and 0.97 for 3D, determined using HiCRep [38] (Figure S2A–C; Table S1). Furthermore, a principal component analysis (PCA) of the individual biological replicates also confirmed their high degree of similarity (Figure S2D). Consequently, we combined both of these datasets for our subsequent analyses, obtaining 151 million and 129 million paired-end reads for the 2D- and 3D-cultured cells, respectively, with an estimated highest map resolution of 40 kb for both [6].

**Figure 2:**
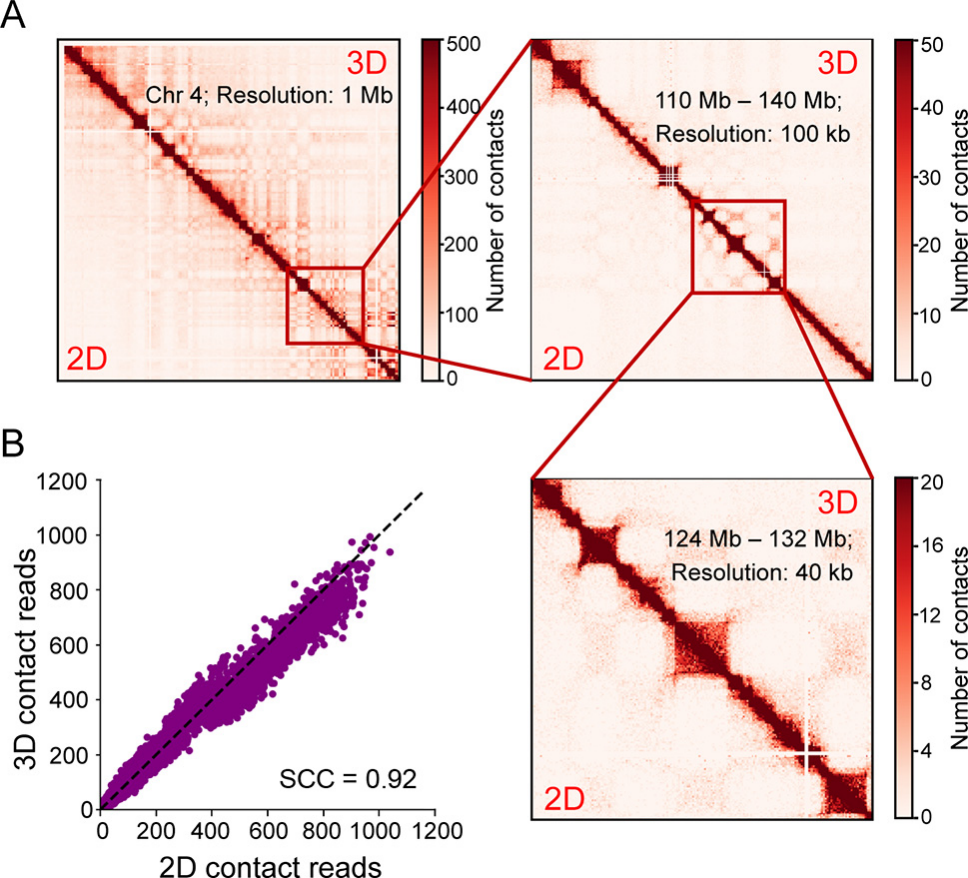
**Hi-C measurements of the 2D- and 3D-cultured AML12 cells** **A.** Heatmaps with increasing resolution as indicated. **B.** Comparison of the contact reads per 40 kb bin. SCC, stratum-adjusted correlation coefficient.

Inspection of the Hi-C heatmaps of these cells revealed clear differences in genomic structure at a longer-length scale (Figure 2A, Figure S2E and F). However, at the highest resolution of 40 kb, the heatmaps in close proximity to the diagonal (reflecting local interactions such as TADs), were virtually indistinguishable (Figure 2A). Consistent with this observation, the Hi-C datasets at this resolution were highly correlated (Figure 2B; SCC = 0.92).

### Comparative examination of the genome structures at the TAD level

**Figure 3:**
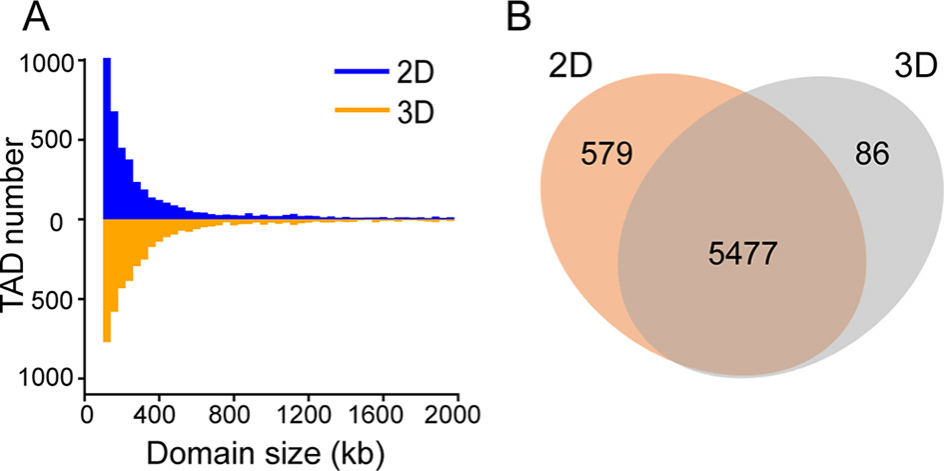
**Characterization of the TAD-level features of the 2D- and 3D-cultured cells** **A.** Size distribution of the TADs annotated from the 40 kb resolution maps. **B.** Venn diagram of the TAD borders of the 2D- and 3D-cultured cells. TAD, topologically associating domain.

We annotated both datasets using the Armatus software [39], and identified a similar number of TADs in both cases (4467 and 4355 TADs in the 2D- and 3D-cultured cells, respectively). The size range and the median size of the identified TADs were also highly consistent under both conditions (median length: 240 kb for the 2D-cultured cells and 280 kb for the 3D-cultured cells; Figure 3A; Table S2). These values are also roughly similar to those of earlier studies of other 2D-cultured cells [6], [8], [40]. Strikingly, the vast majority (>90%) of the TAD borders of these cells overlapped within the limits of our resolution (Figure 3B). Thus, at the TAD level, the genome structures of these hepatocytes are not significantly influenced by the cell culture growth dimension.

### Characterization of the genome structures at the compartment level

**Figure 4:**
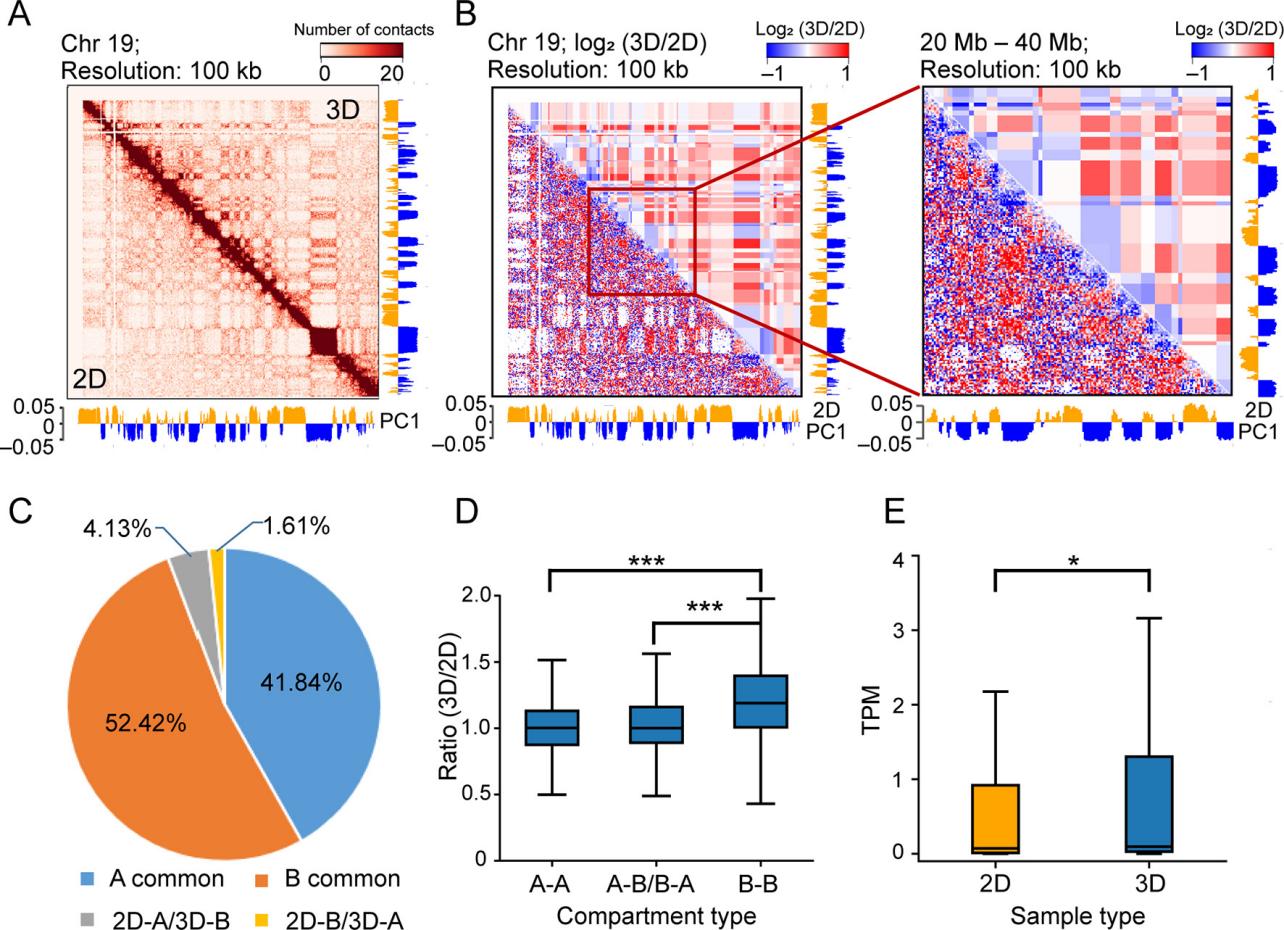
**Comparison of the compartment differences between the 2D- and 3D-cultured cells** **A.** A typical example of a heatmap at the 100 kb resolution, together with the calculated PC1 values used to identify compartments. **B.** Example of the differences in contacts within the compartments. Red and blue represent higher and lower contact frequencies in the 3D-cultured cells, respectively, compared to the 2D-cultured cells. **C.** Pie chart showing the percentages of compartments of a different type between 2D- and 3D-cultured cells. The compartments that have changed types are described according to their designation in the 2D-cultured cells (A or B) changing to that in the 3D-cultured cells (B or A). **D.** Box plot showing the changes in intra-compartment contacts between 2D- and 3D-cultured cells. **E.** Box plot showing the expression changes for genes that are in a B compartment in the 2D-cultured cells but in an A compartment in the 3D-cultured cells. *, *P* < 0.05; ***, *P* < 0.001 (Wilcoxon rank sum test). PC1, first principal component; TPM, transcripts per million.

Since higher-order interactions are also an important aspect of chromosome conformation with functional consequences [41], [42], we further characterized the composition of the A/B compartments of the genomes under 2D and 3D conditions (Figure 4A; Table S3). As shown in Figure 4B, significant differences are apparent at this level, most notably in the compartment type and the magnitude of intra-compartment contact frequency.

**Supplementary Figure S3:**
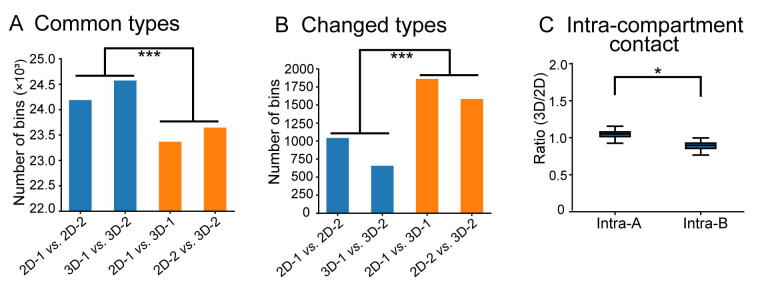
**Significance of compartment-level changes in structure A.** and **B.** Differences in overall compartment size between biological replicates for those compartments that are the same (A) and different (B) type for the 2D- and 3D-cultured cells. ***, P < 0.001, Chi-squared test. **C.** Changes in intra-compartment contacts between 2D- and 3D-cultured cells. ***, *P* < 0.001, Wilcoxon rank sum test.

In particular, we found that 1490 compartments (∼ 6% of all compartments; determined at a 100 kb resolution) are of a different type between the 2D- and 3D-cultured cells (Figure 4C). Since this difference is markedly higher than those between biological replicates (*P* = 1.25 × 10^−198^, Chi-squared test) (Figure S3A and B), the different organization between two conditions is not only significant but also likely consequential. Interestingly, most of these differences (∼ 72%) reflect regions that are A compartments in the 2D-cultured cells but are B compartments in the 3D-cultured cells.

**Supplementary Figure S4:**
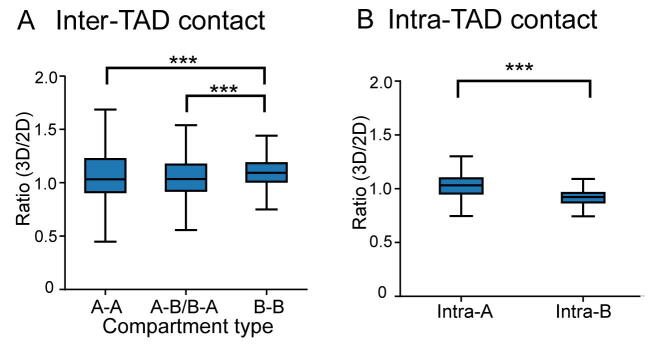
**Significant changes in long-range interactions only for those TADs within B compartments A.** Differences in the longer-range TAD–TAD interactions between 2D- and 3D-cultured cells. ***, *P* < 0.001, Wilcoxon rank sum test. **B.** Differences in the intra-TAD contact frequencies between 2D- and 3D-cultured cells. ***, *P* < 0.001, Wilcoxon rank sum test.

**Supplementary Figure S5:**
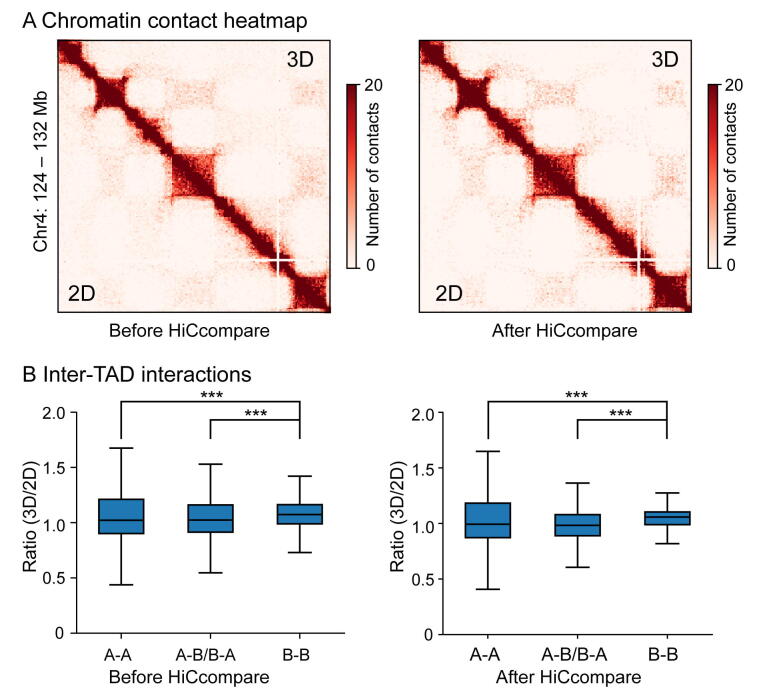
**Examination of potential contributions from condition-specific biases in the 2D/3D data A.** The heatmaps (at a 40 kb resolution) obtained before (left) and after (right) using HiCcompare to normalize the data jointly (2D: SCC = 0.94; 3D: SCC = 0.95). **B.** The significant enrichment of inter-TAD interactions in the B compartments is also similar before (left) and after (right) using HiCcompare.

As for the differences in the magnitude of intra-compartment contact frequency, we found a difference between the A and B compartments. Of all the possible combinations (A-A, A-B/B-A, and B-B) in the 2D/3D-cultured cells, only the B compartments that are in common between 2D- and 3D-cultured cells (B-B) differed in interaction strength, with those of the 3D-cultured cells exhibiting a greater level of intra-compartment contacts than those of the 2D-cultured cells (Figure 4D; Figure S3C). Consistent with this result, there was also a significantly enriched level of inter-TAD contacts, specifically within the B compartments (Figure S4A and B). These results were also observed when we first normalized the 2D and 3D data jointly (Figure S5), indicating that these results were not caused by technical, condition-specific biases in these samples [43]. Thus, while these genomes were essentially identical at a local TAD level, there were considerable differences at the higher-order scale, especially within the B compartments that were in common between 2D- and 3D-cultured cells.

### Comparison between transcriptional and genomic structural changes

**Supplementary Figure S6:**
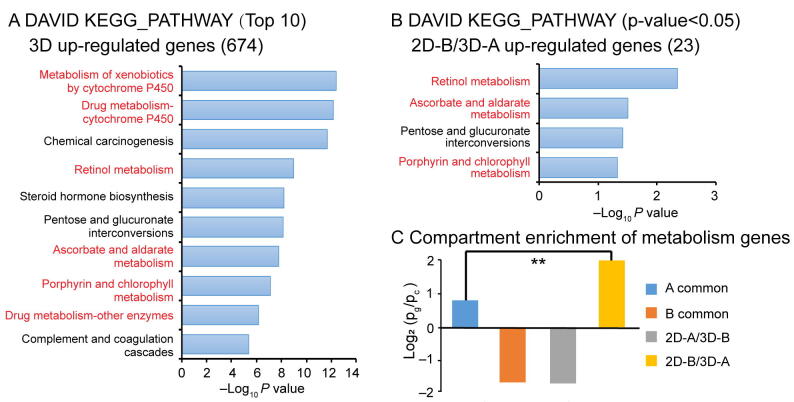
**Gene expression changes between 2D- and 3D-cultured cells A.** Top 10 of the DAVID KEGG_PATHWAYs of the up-regulated genes (*P* < 0.05) in the 3D-cultured cells (DESeq; *P* < 0.05, log2 FC > 2). Colored in red are the metabolic pathways associated with the physiological functions of hepatocytes. **B.** DAVID KEGG_PATHWAYs of the up-regulated genes (DAVID; *P* < 0.05) in the compartments which are B-type in the 2D-cultured cells but A-type in the 3D-cultured cells. **C.** The log2 (pg/pc) of each compartment type, where pg is the ratio of genes associated with “metabolic” functions in each compartment type that are expressed higher in the 3D-cultured cells relative to the total number of “metabolic” genes that are expressed higher in the 3D-cultured cells, and pc is the fraction of each compartment type relative to the total number of compartments. **, *P* < 0.01, Fisher’s exact test.

Based on the commonly observed relationship between transcription levels and genomic structures, particularly at the TAD level, and the high similarity of genomic structures at the TAD level described above, we expected that there might be only a few expression differences between the 2D- and 3D-cultured cells. To verify this hypothesis, we performed an RNA-seq analysis of the cells grown in 2D and 3D in parallel. Contrary to our expectation, more than a thousand genes exhibited significantly different expression in the 3D-cultured cells, including 674 up-regulated genes and 592 down-regulated genes, when compared to the 2D-cultured cells (*P* < 0.05, |log_2_ fold change| > 2). Interestingly, Gene Ontology (GO) analysis showed that the genes that were up-regulated in the 3D-cultured cells were enriched in the terms related to the physiological functions of the hepatocytes (particularly metabolic functions) (Figure S6A; Table S4), in agreement with common expectations [44], [45], [46]. Notably, these genes were not preferentially up-regulated in the 3D-cultured fibroblast cells compared with those cultured in 2D [30].

**Supplementary Figure S7:**
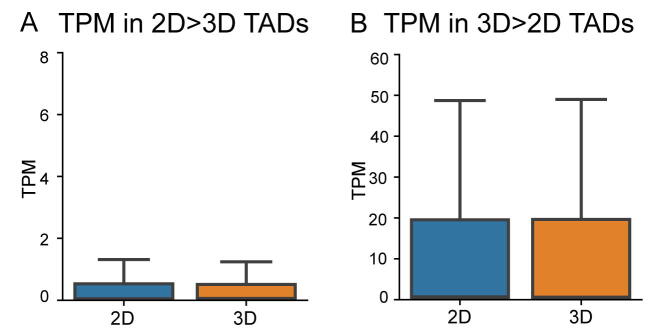
**Relationship between structural changes at the TAD level and expression of the genes contained therein A.** and **B.** Expression level of genes within common TADs where the contact frequency is greater in the 2D-cultured cells than in the 3D-cultured cells (A) or greater in the 3D-cultured cells than in the 2D-cultured cells (B). TPM, transcripts per million.

To examine the possible relationship between gene expression levels and genomic structures at the TAD level, we investigated the expression differences of the genes in the TADs which were shared by the 2D- and 3D-cultured cells but had different contact frequencies. However, we found no significant difference in the expression of the genes in these TADs in the 2D- and 3D-cultured cells (Figure S7A and B).

**Supplementary Figure S8:**
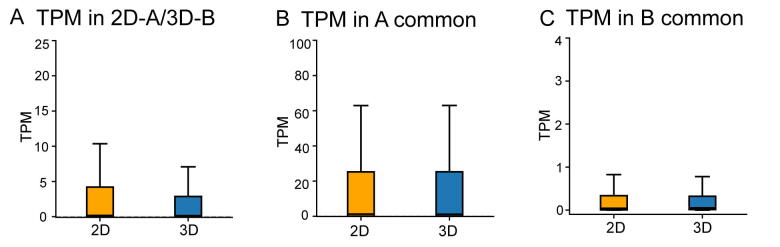
**Absence of any correlation between expression levels and some compartment-level features A.–C.** Expression level of genes within compartments that are A-type in the 2D-cultured cells and B-type in the 3D (A), or A-type in both cells (B), or B-type in both cells (C).

**Supplementary Figure S9:**
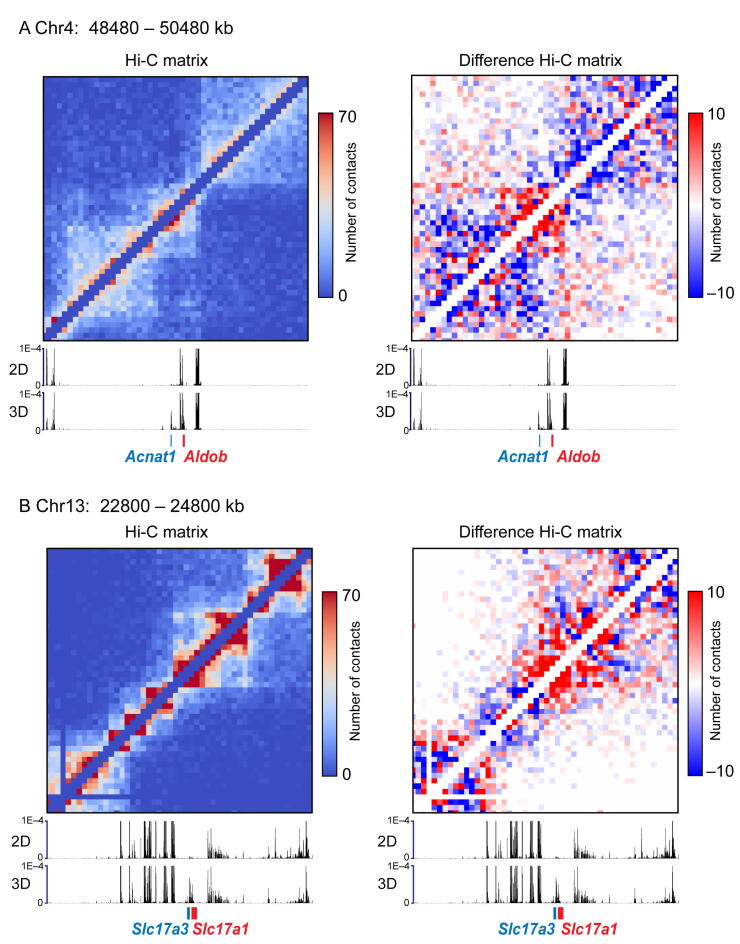
**Examples of genes that are differentially expressed and associated with genomic structure changes** Shown below each Hi-C and difference Hi-C matrix are the RNA-seq reads normalized by the total number of reads. The genes that are differentially expressed are indicated in red and blue.

A similar analysis at the compartment level, however, showed significant differential gene expression within the compartments which were B-type in the 2D-cultured cells but A-type in the 3D-cultured cells (2D-B/3D-A; *P* < 0.05, Wilcoxon rank sum test; Figure 4E). Notably, there was an enrichment of genes that were up-regulated in the 2D-B/3D-A compartments of the 3D-cultured cells, and these genes were associated with the physiological functions of hepatocytes (Figure S6B and C; Table S5). However, examination for the correlation between transcriptional changes and other compartment-level features (*i.e.*, A common, B common, and 2D-A/3D-B) did not identify any enrichment of genes with differential expression (Figure S8A–C). Nonetheless, the up-regulation of the genes associated with the physiological functions of hepatocytes in the 3D-cultured cells appeared to be related to the changes in the compartment-level genomic structures of these cells (Figure S9).

## Discussion

In this study, we investigated the genomic structure and the transcriptome of hepatocytes cultured under 2D and 3D conditions, and examined whether these structural differences correlate with changes in gene expression. This first comparison of the genomic structures of cells grown under these different conditions at the sub-TAD resolution reveals several features of chromosome architecture, particularly with regard to its relationship with gene expression.

First, we find that, at the local TAD level, the architecture of the genomes of the 2D- and 3D-cultured cells is highly similar despite the differences in their transcriptomes. Although the underlying mechanisms driving the formation of TADs are still being uncovered, our observations clearly indicate that they are not exquisitely sensitive to the growth dimension of the cells or to the profound differences in nuclear shape or volume we observed. Furthermore, the absence of any significant correlation between TAD-level structures and the differences in gene expression between 2D- and 3D-cultured cells also indicates that these mechanisms are not highly dependent on, or related to, the transcriptional statuses of genes contained therein. Recent studies have also described a lack of close correlation between changed transcriptomes and differences in Hi-C data at the TAD level [19], [47], [48]. Whether this reflects a change in histone modifications within an essentially similar genomic structure [20], [49], [50], [51] or other mechanisms requires further investigation.

Second, we observe significant differences between 2D- and 3D-cultured cells in higher-order interactions, from TAD–TAD interactions to entire compartments. Some of these differences are associated with differential gene expression between two culture conditions. Furthermore, the correlation between the up-regulation of genes involved in physiological hepatocyte functions in the 3D-cultured cells and the change of genome structure from a B (inactive) compartment in the 2D-cultured cells to an A (active) compartment in the 3D-cultured cells, strongly suggests that some of these structural changes are required to effectuate the more physiological phenotype of the 3D-cultured cells. However, the identification of many structural changes that are not correlated with the changed gene expression suggests that these structural changes could play roles other than gene regulation, an observation that is becoming better appreciated [18], [19], [52].

In conclusion, our work demonstrates the importance of characterizing the genome structure of cells grown under conditions that yield a more physiological phenotype. The genome structure is fundamentally different in cells grown under 2D and 3D conditions, and this difference appears to be consequential to the physiological functioning of the cells. While the ideal sample to investigate are cells within their native tissue, our work shows that culturing cells in 3D provides a readily attainable and highly effective system for this purpose.

## Materials and methods

### Cell culture, synchronization, and crosslinking

AML12 cells (Stem Cell Bank, Chinese Academy of Sciences, Shanghai, China) were cultured in a mix of Dulbecco’s modified Eagle’s medium and Ham’s F12 medium (1:1) containing 10% fetal calf serum and supplied with 1× ITS liquid media supplement [10 μg/ml recombinant human insulin, 5.5 μg/ml human transferrin (substantially iron-free), 5 ng/ml sodium selenite (Catalog No. I3146, Sigma-Aldrich, St. Louis, MO), 40 ng/ml dexamethasone (Catalog No. D4902, Sigma-Aldrich), 100 U/ml penicillin–streptomycin (Catalog No. 11548876, Gibco, Carlsbad, CA)] at 37 °C for 48 h. Then, the cells were dissociated with 0.25% trypsin to suspend them for re-plating. For the 2D cultures, cells were re-plated in 10-cm dishes. For the 3D cultures, cells were grown in Matrigel-embedded 3D cultures in 10-cm dishes. When the cells in the 2D cultures were 50% confluent and the cells in the 3D cultures had grown for 24 h, 1 mM hydroxyurea was added to the medium for 24-h [53] and 36-h incubations for 2D and 3D conditions, respectively. This difference in incubation time with hydroxyurea was chosen owing to the differences in cell-cycle time under these culture conditions. The 2D-cultured cells were cross-linked directly, and then detached from the Petri dish with 1% paraformaldehyde (PFA) for 4 h at 17 °C while mixing in the Petri dish. The PFA was quenched with glycine at a final concentration of 0.125 M for 15 min at room temperature while mixing. The intact spheroids of the 3D-cultured cells were collected with the Cell Recovery Solution (Catalog No. 354253, Corning, NY), and then fixed following the same procedure used for 2D-cultured cells. The cross-linked cells were pelleted by centrifugation, washed with ice-cold PBS, flash-frozen in liquid nitrogen, and finally stored at −80 °C until the preparation of the *in situ* Hi-C libraries.

### Immunofluorescence

For the Lamin B1 staining, the cells were first fixed with 4% PFA at room temperature for 10 min, followed by PFA quenching with glycine at a final concentration of 50 mM. Subsequently, the cells were washed three times with PBS, permeabilized with 0.5% Triton X-100 for 10 min, and then blocked with 5% BSA to reduce non-specific binding. Next, the cells were incubated with the primary antibody, anti-Lamin B1 (1:200; Catalog No. ab16048, Abcam, Cambridge, UK), overnight at 4 °C. The cells were then washed three times with PBS and incubated with the secondary antibody, Donkey anti-Rabbit IgG (H+L) Highly Cross-Adsorbed Secondary Antibody, Alexa Fluor 568 (1:200; Catalog No. A10042, ThermoFisher Scientific, Waltham, MA) for 1 h at room temperature. Finally, the nuclei were stained with DAPI (1 μg/ml). For staining of the plasma membrane, the sample was fixed, permeabilized, and blocked as previously described, and then incubated with Wheat Germ Agglutinin, Alexa Fluor 555 conjugate (1:200; Catalog No. W32464, ThermoFisher Scientific) for 30 min at room temperature. All imaging was performed with confocal fluorescence microscopy (A1Si, Nikon, Japan) at a scan speed of 1/4 frames/s.

### Hi-C library preparation

The cells were thawed on ice and re-suspended into ice-cold lysis buffer [10 mM Tris-HCl pH 8.0, 10 mM NaCl, 0.2% Triton X-100, 1/100 volume of the proteinase inhibitor cocktail (Catalog No. P8340, Sigma-Aldrich)]. Following incubation on ice for 30 min, the nuclei were pelleted and washed with ice-cold lysis buffer. After washing with 1.2× cutsmart buffer (Catalog No. B7204, NEB, Ipswich, MA), the nuclei were re-suspended with 1.2× cutsmart buffer supplemented with 0.1% SDS and incubated for 1 h at 65 °C. After, the nuclei were incubated with 1% Triton X-100 for 15 min at 37 °C, and then digested overnight at 37 °C with 100 U of restriction endonuclease *Mbo*I (5 U/μl; Catalog No. R0147, NEB) with slow rotation.

The nuclei were pelleted and washed twice with 1× NEBuffer 2 (Catalog No. B7002, NEB). The nuclei were then re-suspended with 1× NEBuffer 2 containing 0.015 mM dCTP, 0.015 mM dGTP, 0.015 mM dTTP, 0.015 mM biotin-14-dATP, and 5 U of Klenow enzyme (Catalog No. M0210, NEB). The mixture was incubated at 37 °C for 2 h with slow rotation. Next, the nuclei were harvested and re-suspended in the ligation master mix [1× T4 ligase buffer (NEB), 0.1 mg/ml BSA, 40 U/μl of T4 ligase (Catalog No. B0202, NEB) in ddH_2_O], followed by incubation at 25 °C for 4 h and 16 °C for 8 h. The crosslinking was then reversed by incubating overnight at 65 °C with proteinase K (1 μg/μl). The DNA was extracted with phenol–chloroform and purified with ethanol precipitation. Then, the RNA was removed by RNase A (1 μg/μl), and the DNA was sheared with Covaris M220. The DNA fraction in the size range of 300–500 bp was collected with Agencourt AMPure XP beads (Catalog No. A63881, Beckman Coulter, Brea, CA).

The DNA was end-repaired and ‘A’ tailed with the “NEBNext End Pre” module, and adapters were ligated with the “Adaptor ligation” module in the NEBNext Ultra DNA Library Prep Kit for Illumina (Catalog No. E7370, NEB) according to the manufacturer’s instructions. Subsequently, biotin-mediated pull-down of the ligation products was performed as previously described [6] with minor modification. Then, the DNA suspension was transferred into a PCR tube and PCR amplified with index primers, universal PCR primers, and the NEBNext High Fidelity 2× PCR Master Mix. The PCR reactions were performed following the manufacturer’s instructions of the NEBNext Ultra DNA Library Prep Kit for Illumina (Catalog No. E7370, NEB). The concentrations of the Hi-C libraries were determined using the Qubit dsDNA HS Assay, and the quality was measured by Agilent 2100 DNA 1000 HS Kit. Lastly, the high-quality libraries were sequenced using an Illumina X-ten platform with 150 bp paired-end reads.

### Hi-C data processing

We mapped all Hi-C reads to the mm10 mouse reference genome using Bowtie 2 (v2.2.9) iteratively as described [37]. For each end of the Hi-C reads, we first mapped an outermost length of 30 bp, and if this length of region failed to map uniquely, we included an additional 20 bp in the next round of mapping. This procedure continued until the mapping length reached the full read length of 150 bp. Read pairs with mapping quality (MAPQ) larger than 30 for each end were kept. Then, the reads mapped to the same restriction fragment and the dangling reads having a separating genomic distance shorter than 500 bp were removed. We generated Hi-C contact matrices at 1 Mb, 100 kb, or 40 kb resolutions for each chromosome and normalized using ICE [54]. To test the validity of our data, we calculated the SCC [38] between the biological replicates of the 2D- and 3D-cultured cells, respectively. For comparison, we selected the same number (35 M) of intra-chromosomal reads for each replicate of the 2D- and 3D-cultured cells. The fractions of *trans*-interactions among the valid reads for the 2D and 3D datasets were ∼ 32% and ∼ 43%, respectively, within the range (27%–57%) of several recently published Hi-C datasets [20], [55], [56], [57], [58], [59]. Furthermore, these percentages were highly consistent between individual biological replicates (31%/32% for 2D and 42%/43% for 3D).

### Annotation of the compartments and TADs

We annotated the compartments at a 100 kb resolution as previously described [7]. Specifically, we performed a PCA of the normalized contact matrix for each chromosome, and assigned the signs of the first principal component (PC1) to different types of compartments. To determine the signs of the PC1, we calculated the Pearson correlation coefficient (PCC) between the eigenvectors and the read densities of RNA-seq of the corresponding regions. If the PCC was negative, the eigenvector was multiplied by −1. Positive values of the PC1 defined A-type compartments and negative ones defined B-type.

To reduce the bias introduced by the differences in sequencing depth, we normalized the total contact numbers of each chromosome in the 2D and 3D datasets to the same depth before annotating the domains. After the depth normalization, we annotated the domains using the software Armatus v1.0 [39], [60] with the gamma parameter set to 0.8.

### Calculation of the compartment–compartment and TAD–TAD contact enrichment

For each pair of compartments and TADs, we calculated the average contact frequencies of all pairs of bins with gap bins excluded. We removed the compartment and TAD pairs if the number of gap bin pairs took more than 50% of the total bin pairs within the corresponding compartments or TADs. The average contact frequencies of TAD–TAD or compartment–compartment pairs between 2D- and 3D-cultured cells were then calculated.

### RNA-seq library construction

Total RNA was extracted from 3 million cells using Trizol Reagent, and the quality was assessed using Agilent Bioanalyzer 2100. The RNA-seq libraries were prepared using the KAPA Stranded mRNA-seq Kit (Catalog No. 07962193001, Roche, Basel, Switzerland) following the manufacturer’s instructions. The quality of the libraries was measured by the Qubit fluorometer and Agilent Bioanalyzer 2100. The libraries were then sequenced with the Illumina X-ten platform (2 × 150).

### RNA-seq data analysis and functional annotation

We removed adapters and low-quality reads using cutadapt (v1.8.3) [61] and Trimmomatic (v0.36) [62] with default parameters. The remaining valid paired reads were mapped to the GRCm38 mouse reference genome by HISAT2 (v2.0.5) [63] with options “--rna-strandness RF --no-softclip”. We next assembled and quantified the transcripts using Stringtie (v1.3.4) [64] with the Mus_musculus.GRCm38.93.chr GTF file downloaded from the Ensembl database. The read counts of the detected genes were extracted from the output files of Stringtie through the Python script “prepDE.py” provided online (https://ccb.jhu.edu/software/stringtie/dl/prepDE.py). To evaluate the difference in gene expression between 2D- and 3D-cultured cells, we used the R package “DESeq2” [65] to calculate the fold change of the transcription levels and assigned a statistical significance to each pair of genes. Genes with |log_2_ fold change| > 2 and *P* < 0.05 were identified as differentially expressed genes. The GO terms for the gene functional classification were determined using DAVID (v6.8) (https://david.ncifcrf.gov/gene2gene.jsp).

## Data availability

The Hi-C and RNA-seq datasets in this study have been deposited at the Gene Expression Omnibus (GEO: GSE136307) that are publicly accessible at https://www.ncbi.nlm.nih.gov/geo, and in the Genome Sequence Archive [66] at the National Genomics Data Center, Beijing Institute of Genomics, Chinese Academy of Sciences / China National Center for Bioinformation (GSA: CRA002599) that are publicly accessible at https://ngdc.cncb.ac.cn/gsa.

## CRediT author statement

**Xin Liu:** Investigation, Methodology, Writing - original draft. **Qiu Sun:** Software, Formal analysis, Data curation, Writing - review & editing. **Qi Wang:** Investigation, Methodology, Writing - review & editing. **Chuansheng Hu:** Formal analysis. **Xuecheng Chen:** Investigation. **Hua Li:** Formal analysis. **Daniel M. Czajkowsky:** Conceptualization, Formal analysis, Writing - original draft. **Zhifeng Shao:** Conceptualization, Methodology, Writing - original draft. All authors have read and approved the final manuscript.

## Supplementary material

Supplementary Table S1 **Filter results and SCC results of the sequencing data**

Supplementary Table S2 **TADs annotation results**

Supplementary Table S3 **Compartments annotation results**

Supplementary Table S4 **DAVID GO terms and genes associated with “metabolic” functions of 3D up-regulated genes**

Supplementary Table S5 **Biological function of select genes up-regulated in the 3D-cultured cells**
